# Inducible mutant huntingtin expression in HN10 cells reproduces Huntington's disease-like neuronal dysfunction

**DOI:** 10.1186/1750-1326-4-11

**Published:** 2009-02-09

**Authors:** Andreas Weiss, Ana Roscic, Paolo Paganetti

**Affiliations:** 1 Neuroscience Discovery, Novartis Institutes for BioMedical Research, Novartis Pharma AG, Basel, Switzerland

## Abstract

**Background:**

Expansion of a polyglutamine repeat at the amino-terminus of huntingtin is the probable cause for Huntington's disease, a lethal progressive autosomal-dominant neurodegenerative disorders characterized by impaired motor performance and severe brain atrophy. The expanded polyglutamine repeat changes the conformation of huntingtin and initiates a range of pathogenic mechanisms in neurons including intracellular huntingtin aggregates, transcriptional dysregulation, energy metabolism deficits, synaptic dystrophy and ultimately neurodegeneration. It is unclear how these events relate to each other or if they can be reversed by pharmacological intervention. Here, we describe neuronal cell lines expressing inducible fragments of normal and mutant huntingtin.

**Results:**

In HN10 cells, the expression of wild type and mutant huntingtin fragments was dependent on the induction time as well as on the concentration of the RheoSwitch^® ^inducing ligand. In order to analyze the effect of mutant huntingtin expression on cellular functions we concentrated on the 72Q exon1 huntingtin expressing cell line and found that upon induction, it was possible to carefully dissect mutant huntingtin-induced phenotypes as they developed over time. Dysregulation of transcription as a result of mutant huntingtin expression showed a transcription signature replicating that reported in animal models and Huntington's disease patients. Crucially, triggering of neuronal differentiation in mutant huntingtin expressing cell resulted in the appearance of additional pathological hallmarks of Huntington's disease including cell death.

**Conclusion:**

We developed neuronal cell lines with inducible expression of wild type and mutant huntingtin. These new cell lines represent a reliable *in vitro *system for modeling Huntington's disease and should find wide use for high-throughput screening application and for investigating the biology of mutant huntingtin.

## Background

Huntington's disease (HD) is an inherited, autosomal-dominant neurodegenerative disorder whose main clinical symptoms include chorea, cognitive decline and weight loss [1]. Disease onset occurs normally at mid-age followed by a relentless disease progression and premature death 10 to 20 years after appearance of clinical symptoms [2]. HD is caused by a mutated and expanded CAG repeat in the huntingtin gene (*htt*) resulting in an elongated polyglutamine (polyQ) stretch in the mutant huntingtin protein (Htt) [3].

Proteolytic cleavage of full-length mutated Htt could represent the pathogenic rate-limiting step [4] which produces aggregation-prone amino-terminal Htt fragments carrying the expanded polyQ repeat [5]. These fragments are toxic *in vitro *and *in vivo *through a gain of function mechanism but the causes for neuronal dysfunction initiated by mutant Htt are not fully understood [6,7]. Mutant Htt toxicity correlates with the length of the polyQ repeat and the amount of mutant Htt fragments accumulating in the cells, whereby shorter Htt fragments are more pathogenic than longer fragments or full-length Htt [5,8-11]. Different pathogenic mechanisms are discussed, including dysregulation of transcription [12], abnormalities in the ubiquitin/proteasome system [13], impaired axonal trafficking, microtubule destabilization [14,15] and mitochondrial dysfunction [16]. One of the earliest events is the typical transcription dysregulation signature displayed in cellular and animal HD models of the disorders as well as in the HD brain [12]. Transcription factors such as specificity protein 1 (SP1) [17] and cAMP response element binding protein (CREB) [18] interact with mutant Htt and are sequestered into aggregates, resulting in transcription abnormalities of their target genes.

Cellular models of HD are essential for studying the biology of mutant Htt. Non-neuronal primary cells from HD patients have been used to study CAG-repeat instability [19] or calcium homeostasis [20]. PC12-cell based models of Htt toxicity have been used for drug screens [21]. Mutant Htt leads to neurite degeneration and induction of apoptosis in murine primary neuronal cultures [22,23]. Immortalized striatal neurons showed increased vulnerability to mitochondrial toxins and impaired mitochondrial complex II function in presence of stable mutant Htt expression [24,25]. Such models based on primary neurons cultures or Htt expressing cell lines greatly improved our understanding of HD pathogenesis. Nevertheless, they also present some drawbacks for use in drug discovery such as the limited use of primary neuronal cultures for high-throughput screening or the adaptive changes expected in cells chronically exposed to stably expressed mutant Htt. We opted to address some of these aspects by generating inducible HN10 neuronal lines. HN10 cells resist several treatments such as cDNA transfection and compound treatment and can be easily differentiated into neuron-like post-mitotic cells [26,27]. Upon induction of Htt expression and differentiation into post-mitotic neurons, HN10 cells develop a number of HD-like phenotypes. Our cell lines can be used for chemical and genetic high-throughput screenings, represent models for dissecting the mechanism of action of drug candidates and allow for assessing the role of novel targets in neuroprotection.

## Materials and methods

### HN10 cells culturing conditions

Dividing HN10 cells were propagated in proliferating culture medium: high-glucose DMEM (Invitrogen #31966-021), 10% fetal calf serum, penicillin, geneticin, hygromycin and streptomycin and maintained at 37°C in 5% CO_2_. Medium replacement and passaging of the cells occurred every 2–3 days. In order to induce cell differentiation, proliferating cells were harvested, resuspended in proliferating culture medium and plated at 20% confluency on dishes pre-coated with 20 μg/μl laminin (SIGMA #L2020). The cells were then cultured one day later in differentiating medium: serum-free high-glucose DMEM, neuronal supplements [28] and 45 μM retinoic acid (SIGMA #R2625) for up to 7 days.

### Production of stable inducible cell clones

HN10 cells were transfected with the RheoReceptor pNEBR-R1 plasmid (RheoSwitch Mammalian Inducible Expression System, New England Biolabs) using Lipofectamine™ 2000 (Invitrogen #11668-019). Transfected cells were counted and seeded in 96-well plates. Dilution series were made in a way to obtain single clones in the wells with the highest cell dilutions. Transfected cells were selected using 1 mg/ml Geneticin (Invitrogen #10131-027). After 2 weeks of selection, more than 30 clones were harvested and further expanded in a 24-well plate. Twenty one clones were selected based on similarity to the parental HN10 cell line in terms of morphology, growth rate and ability to differentiate. Successful integration of the RheoReceptor was confirmed by transient transfection with the reporter plasmid pNEBR-X1 expressing Gaussia luciferase and treatment for two days in the presence of 500 nM RSL1 inducer. The receptor-clone #14, which displayed the best luciferase induction ratio and no basal expression, was selected for production of Htt inducible cell lines expressing the amino-terminal Htt fragments 25Q Htt-exon1 (cell line Exon1-Q25-S), 72Q Htt-exon1 (Exon1-Q72-V), 25Q Htt-857 (aa857-Q25-B) and 72Q Htt-857 (aa857-Q72-B) subcloned in the pNEBR-X1Hygro plasmid.

### Subcellular fractionation

The HN10 cell line Exon1-Q72-V was cultured in the presence or absence of 500 nM RSL1 for two days. Cells were then washed with PBS and nuclear and cytosolic fractions were obtained as described [29]. Purity of the nuclear and cytosolic fractions was verified by western blot using an antibody against a protein marker for the cytoplasma (tubulin; abcam antibody #ab28037-100; dilution 1:4000) or for the nucleus (histone H3; Chemicon antibody #MAB052; 1:2000).

### Immunostaining

HN10 cells were fixed in 4% formaldehyde and stained with 1:500 anti-Htt antibody 2B7 (custom production by NanoTools, Freiburg, Germany), 1:100 anti-Htt mEM48 antibody (Millipore, MAB 5374) or 1:1000 anti-polyglutamine antibody m1C2 (Millipore, MAB 1574). Cell nuclei were visualized with 1:20000 Hoechst nuclear stain (Invitrogen #33258). Standard immunohistochemistry protocols and fluorescence microscopy were used.

### Western Blot and AGERA

Soluble and aggregated mutant Htt fragments were determined by western blot or AGERA as described [11].

### Real-time PCR

The HN10 clone 72Q Htt-exon1 was cultured under proliferating or differentiating conditions with or without induction of Htt expression in a 24 well plate (Becton Dickinson #353047). Total RNA was isolated with the RNeasy Mini Kit (Qiagen). Real-time PCR was performed using customized TaqMan^® ^microfluidic card arrays (Applied Biosystems) according to the manufacturer protocol. Eight different samples were analyzed simultaneously against 15 probes and one 18S normalization control probe. First-Strand cDNA synthesis was done with 100 to 150 ng RNA according to the manufacturer protocol (Invitrogen, SuperScript III Platinum #11732-088). PCR amplifications in the microfluidic card were performed using a solution made of 30 μl cDNA, 50 μl 2× Platinum qPCR SuperMix-UDG with ROX (Invitrogen #11744-500) and 100 μl DEPC water. After two centrifugations of 1000 g for 1 min, microfluidic cards were sealed and analyzed after an incubation at 50°C for 2 min and at 94.5°C for 2 min. Amplification parameters corresponded to 45 cycles at 97°C for 30 sec and 59.7°C for 1 min. Ct values were normalized against the 18S calibration control.

### Protein determination and aconitase measurements

For protein and aconitase measurements, HN10 cells were cultured on 24-well plates (Becton Dickinson #353047) with a starting plating density of 34 000 cells/well. After 1, 2 and 3 days of cell culture, cells were washed three times with PBS and protein content in each well was measured with the BCA™ Protein Assay Kit (Perbio, #23227). The aconitase activity assay was adapted from published methods [30,31]. Briefly, cells were washed with 100 μl/well PBS followed by addition of 100 μl/well aconitase buffer: 30 mM sodium citrate, 0.5 mM MnCl_2_, 0.2 mM NADP, 2 U/ml isocitrate dehydrogenase, 1% Triton in 50 mM Tris adjusted to pH 7.5. Kinetic measurements over the first 30 min were done at 37°C using a Fluoroskan (Thermo Scientific, Fluoroskan Ascent FL) with excitation at 355 nm and emission at 460 nm. Aconitase activity was determined by linear regression of the linear part of the curve.

### Caspase 3/7 activity

HN10 cells were cultured under differentiating conditions with or without inducer for up to 5 days on opaque 96-well plates (Becton Dickinson #353296). Total caspase 3/7 activity per well was determined using the caspase-Glo 3/7 Assay (Promega, #G8091) as recommended by the manufacturer using a RUBYstar Reader (BMG Labtech).

### Neurite quantification

Analysis of the neurite network was performed with a LI-COR Biosciences In-Cell Western™ assay. HN10 cells were cultured under differentiating conditions for up to 6 days on clear bottom 96-well plates (Becton Dickinson #353072). During this incubation time, HN10 cells formed an extensive neurite network. After fixation with 4% paraformaldehyde, neurites were immunostained with an anti-α-tubulin antibody (1:1000, abcam, #ab28037-100) and IRDye 800CW secondary antibody (LI-COR Biosciences). Total amount per well of tubulin was determined with an Odyssey Imager (LI-COR Biosciences) and normalized against number of nuclei (DRAQ5™, Biostatus Limited).

## Results

### Inducible expression of Htt in the neuronal HN10 cell lines

**Figure 1:**
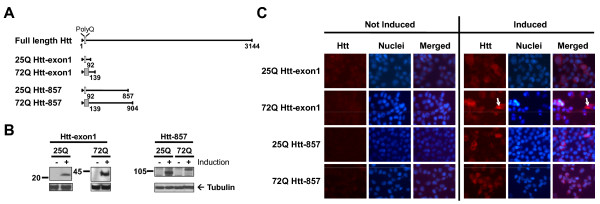
**Inducible expression of wild-type and mutant huntingtin fragments in clonal HN10 cell lines**. **A**: Scheme of normal (25Q) and mutant (72Q) Htt fragments used for creation of inducible HN10 cell lines. **B**: Induction of Htt expression with 500 nM RheoSwitch^® ^Ligand (RSL1) normalized for tubulin content in cell lysates demonstrated by western blot analysis. No basal expression is detected in non-induced cells. **C**: Expression of induced Htt shown by fluorescent immunocytochemistry of Htt cell lines treated in the presence or absence of inducer. Five days of induction with 500 nM RSL1 results in the appearance of Htt inclusions specifically in a subset of 72Q Htt-exon1 cells (white arrows).

*In vitro *models facilitate the dissection of Htt toxicity. Optimally, these cellular models will recapitulate HD-like pathological hallmarks. We generated four stable neuronal HN10 clone lines with inducible expression of short (Htt-exon1) or long (Htt-857) amino-terminal Htt fragments carrying either normal (25Q) or mutant (72Q) polyQ lengths (Fig. 1A). All cell lines displayed no basal expression of the fragments, whereas inducible Htt expression was demonstrated by western blot analysis of cell lysates (Fig. 1B) and immunohistochemistry, whereby a subset of 72Q Htt-exon1 cells formed cellular aggregates within few days after induction (Fig. 1C).

**Figure 2:**
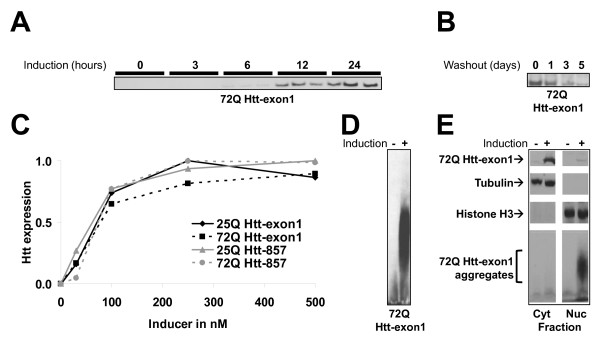
**Regulated expression of 72Q Htt-exon1 and aggregation formation**. **A**: Addition of 500 nM RSL1 inducer to the culture medium leads to a time dependent increase in 72Q Htt-exon1 expression first detectable after 6 h of treatment (western blot of biological triplicates). **B**: Time-dependent disappearance of 72Q Htt-exon1 in HN10 cells upon removal of the RSL1 inducer from the culture medium. **C**: Dose dependent Htt expression as a function of RSL1 concentration in the culture medium of 25Q Htt-exon1, 72Q Htt-exon1, 25Q Htt-857 and 72Q Htt-857 cell lines (western blot quantification). **D**: AGERA blot of 72Q Htt-exon1 cell lysates reveals significant accumulation of Htt aggregates after induction of Htt expression but not in non-induced cells. **E**: Subcellular fractionation of cytoplasma and nuclei demonstrate the presence of abundant amounts of soluble Htt in the cytoplasma of the HN10 cells. In contrast, a minor pool of soluble mutant Htt and all aggregated 72Q Htt-exon1 were detected in the nuclei.

Htt fragments were first detected 6 h upon induction and reached maximal expression at 24 h (Fig. 2A). Removal of the inducer from the culture medium caused a time-dependent disappearance of the Htt fragments (Fig. 2B) demonstrating expression reversibility and rapid Htt degradation. Quantification of Htt by western blot revealed dose-dependent increase in expression as a function of the amount of inducer (Fig. 2C). Supporting the immunohistochemical observation, aggregate formation in the 72Q Htt-exon1 cells was confirmed by AGERA (Fig. 2D). No detectable aggregates were found in cells expressing 72Q Htt-857, 25Q Htt-857 or 25Q Htt-exon1 fragments (data not shown).

In cellular and animal models of HD mutant huntingtin normally accumulates in nuclear aggregates [23,32] leading to transcription abnormalities. To assess the intracellular distribution 72Q Htt-exon1 protein in HN10 cells, we isolated the nuclei of HN10 cells. The purity of the nuclear fraction was demonstrated by Western blot using histone H3 as a protein marker for the nucleus and the cytoplasmic marker tubulin (Fig. 2E). Using this protocol, we demonstrated distribution of the soluble pool of mutant Htt in both the cytoplasmic and the nuclear fraction, whereby mutant Htt appeared predominantly localized in the cytoplasma. In contrast, mutant huntingtin aggregates accumulated exclusively in the nuclei of HN10 cells after two days of induction (Fig. 2E).

**Figure 3:**
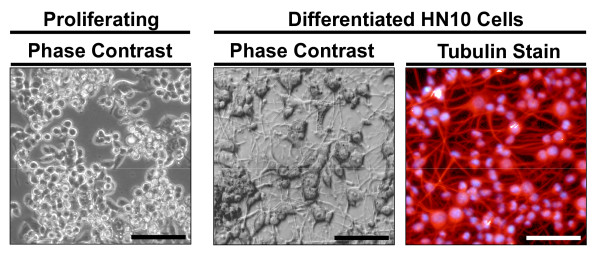
**HN10 cells grown under proliferating and differentiated conditions**. Adjusting the culture conditions results in differentiation of HN10 cells in a characteristic neuronal morphology. Representative phase-contrast micrographs and a fluorescent image of HN10 cells after immunocytochemistry staining with a tubulin-specific antibody (red channel) and Hoechst nuclear marker (blue channel). A dense neurite network is visible at 7 days of HN10 cell differentiation (bar = 100 μm).

All subsequent experiments were aimed at characterizing the 72Q Htt-exon1 cell line as it expresses a short amino-terminal Htt fragment, which is expected to display prominent toxicity. Importantly, mutant Htt-dependent impairment of cell viability is most pronounced in post-mitotic neurons [32-35]. In order to differentiate HN10 cells, we applied culturing conditions optimized for primary neurons that indeed induced rapidly a neuron-like morphology (Fig. 3). Under these conditions, differentiated HN10 cells displayed neuronal properties [26].

### Mutant Htt causes transcription abnormalities

We examined the consequence of mutant Htt induction on transcription dysregulation of representative genes that exhibit SP1 or CREB control elements in their respective promoters.

**Figure 4:**
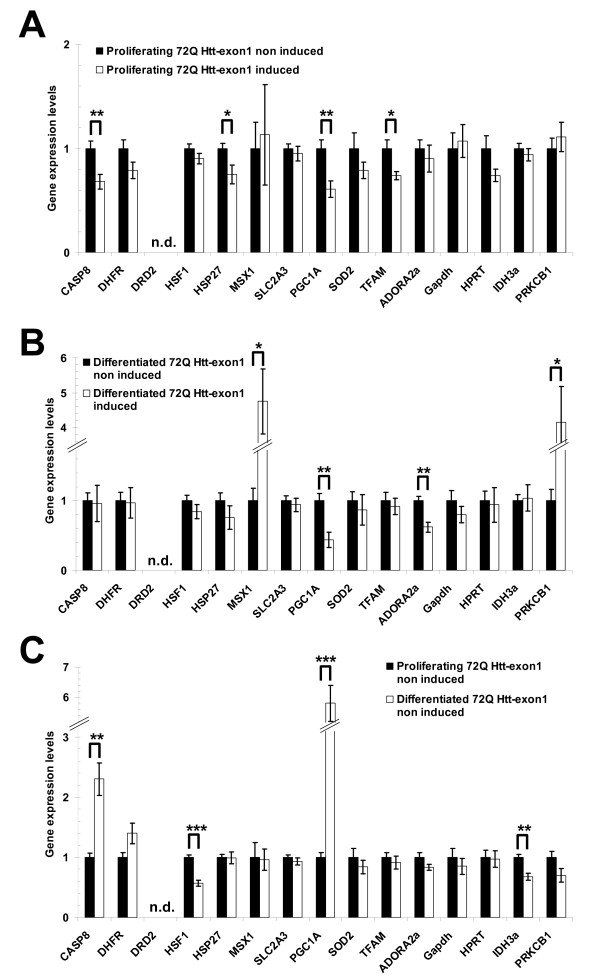
**Transcriptional abnormalities in proliferating and differentiated HN10 cells in the presence or absence of mutant huntingtin**. **A**: mRNA determinations by real-time PCR of a selected panel of genes in mutant 72Q Htt-exon1 expressing and control proliferating HN10 cells. **B**: Same as in A using differentiated, non-dividing HN10 cells. **C**: Effect of differentiation of HN10 cells on transcription of the same genes. All graphs: n = 3, * p < 0.05*, ** p < 0.01, *** p < 0.001.

First, actively dividing HN10 cells expressing 72Q Htt-exon1 where compared to non-induced cells. Among the 15 mRNA analyzed, four genes showed a significant down-regulation of transcription in the presence of mutant Htt (caspase 8, HSP27, PGC-1α and TFAM; Fig. 4A). The strongest effect was found for the peroxisome proliferator-activated receptor-gamma coactivator 1α (PGC-1α), a CREB and Sp1 dependent master regulator of mitochondriogenesis and of the reactive oxygen protective pathway [36]. A difference in PGC-1α expression is a critical feature of a cell model of the disorder as it has been reported to be robustly down-regulated in medium spiny neurons in HD [37,38]. Caspase 8 and the heat shock protein HSP27 are under the control of the Sp1 element, whereas the mitochondrial transcription factor TFAM is under the control of CREB. Another prominent hallmark gene in the transcription signature of HD is the dopamine receptor DRD2, whose expression was not detected in the hippocampal HN10 cells. The fact that only a subset of genes was affected may highlight early and possibly more relevant transcription abnormalities occurring in cells after exposure to mutant Htt.

In differentiated HN10 cells, induction of 72Q Htt-exon1 expression was also accompanied by transcription dysregulation of four genes (Fig. 4B), whereby two genes were down-regulated (PGC-1α and the adenosine A2a receptor ADORA2a) and two genes were up-regulated (MSX1 and PRKCB1) when compared to cells lacking mutant Htt. Abnormalities in ADORA2a were reported to be associated with HD [39,40]. Msh-like 1 (MSX1) homeobox has been reported to represent a candidate modifier of HD age-of-onset [41], whereas protein kinase C β1 (PRKCB1) has been shown to be dysregulated in HD [42]. Transcription dysregulation was more prominent in differentiated HN10 cells than in proliferating cells, possibly because of increased Htt accumulation in post-mitotic cells or because neurons are more susceptible to mutant Htt toxicity.

Mutant Htt expression caused transcription dysregulation of different subsets of genes (with the exception of PGC-1α) when comparing proliferating and differentiated HN10 cells. This apparent discrepancy may result from differentiation-dependent changes in overall gene transcription. To explore this possibility in more details, we compared gene expression in proliferating versus differentiated HN10 in the absence of mutant Htt (Fig. 4C). Not surprisingly, cell differentiation had indeed significant effects on gene transcription with caspase 8, heat-shock factor 1 (HSF1), PGC-1α and NAD(+)-dependent isocitrate dehydrogenase subunit 3 (IDH3) being significantly changed upon differentiation. Up-regulation of PGC-1α may reflect the increased energetic demand of neuronal cells due to extensive neurite formation and for maintaining the membrane potential.

### Mutant Htt expression induces mitochondrial abnormalities and impairs cell viability

**Figure 5:**
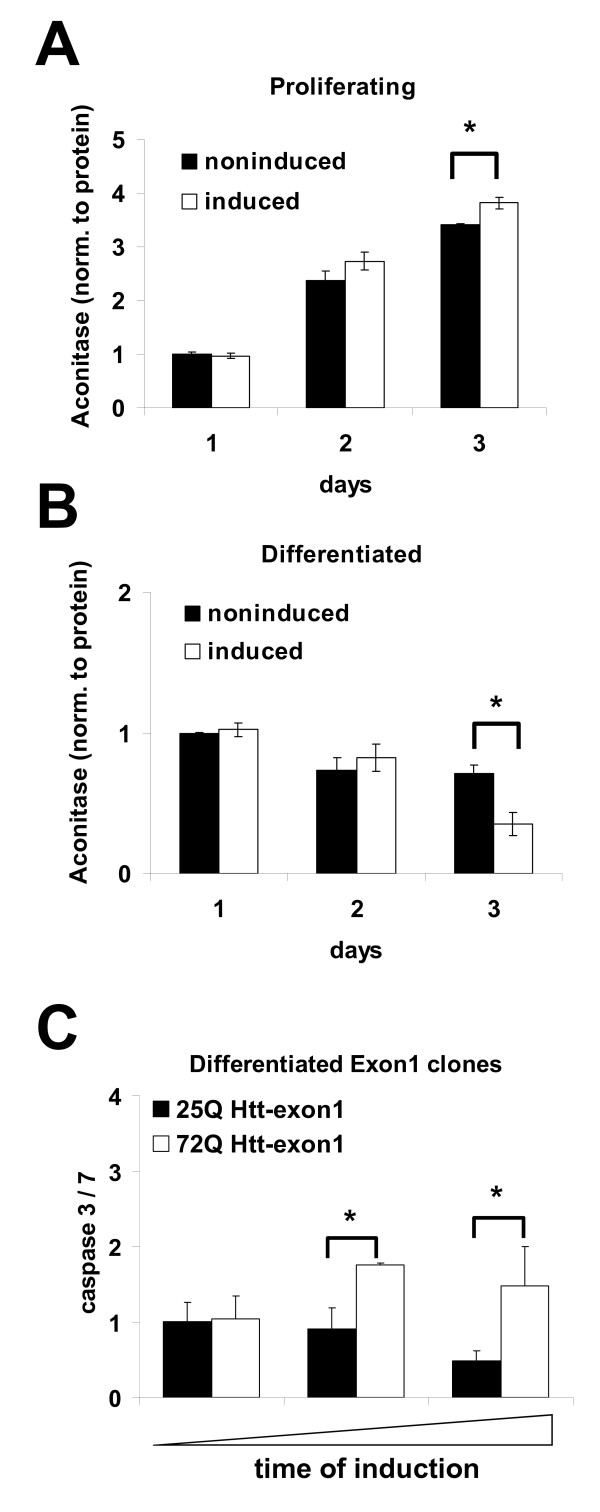
**Impaired aconitase activity and increased caspase 3 activation resulting from 72Q Htt-exon1 expression in differentiated cells**. Aconitase activity determination in cell lysates one and two days after induction of mutant Htt expression with 500 nM RSL1 in proliferating (**A**) and differentiated (**B**) 72Q Htt-exon1 cells (white bars) compared to non-expressing cells (black bars). **C**: Increase in caspase 3/7 activity in cell lysates after induction of 72Q Htt-exon1 expression with 500 nM RSL1 in differentiated HN10 cells (white bars). Expression of the wild-type 25Q Htt-exon1 decreases caspase 3/7 activity (black bars). All graphs: n = 3, * p < 0.05, ** p < 0.01.

Dysregulation of PGC-1α and TFAM mRNA levels were indicative of possible abnormalities in mitochondrial function. Using aconitase activity as a marker for mitochondrial oxidative damage [31,43,44], we examined the influence of 72Q Htt-exon1 expression on aconitase activity in proliferating or differentiated HN10 cells. Three days after induction of mutant Htt expression, we found a significant mitochondrial impairment in differentiated cells (Fig. 5B) but not in proliferating cells (Fig. 5A) when compared to non-induced control HN10 cells.

Mitochondrial dysfunction may lead to apoptosis. Measuring caspase 3/7 induction as a proapoptotic marker of compromised cell viability, we observed increased caspase 3/7 activity in differentiated HN10 cells in the presence of 72Q Htt-exon1 (Fig. 5C) and 72Q Htt-857 (data not shown). To confirm that the effects are a direct result of the elongated polyQ stretch, we analyzed the consequence of wild-type Htt. In contrast to the effects observed upon induction of 72Q Htt-exon1, the expression of 25Q Htt-exon1 resulted in decreased caspase 3/7 activity, confirming the neuroprotective properties reported for wild-type Htt (Fig. 5C) [45].

### Neurodegenerative properties of mutant Htt in HN10 cells

**Figure 6:**
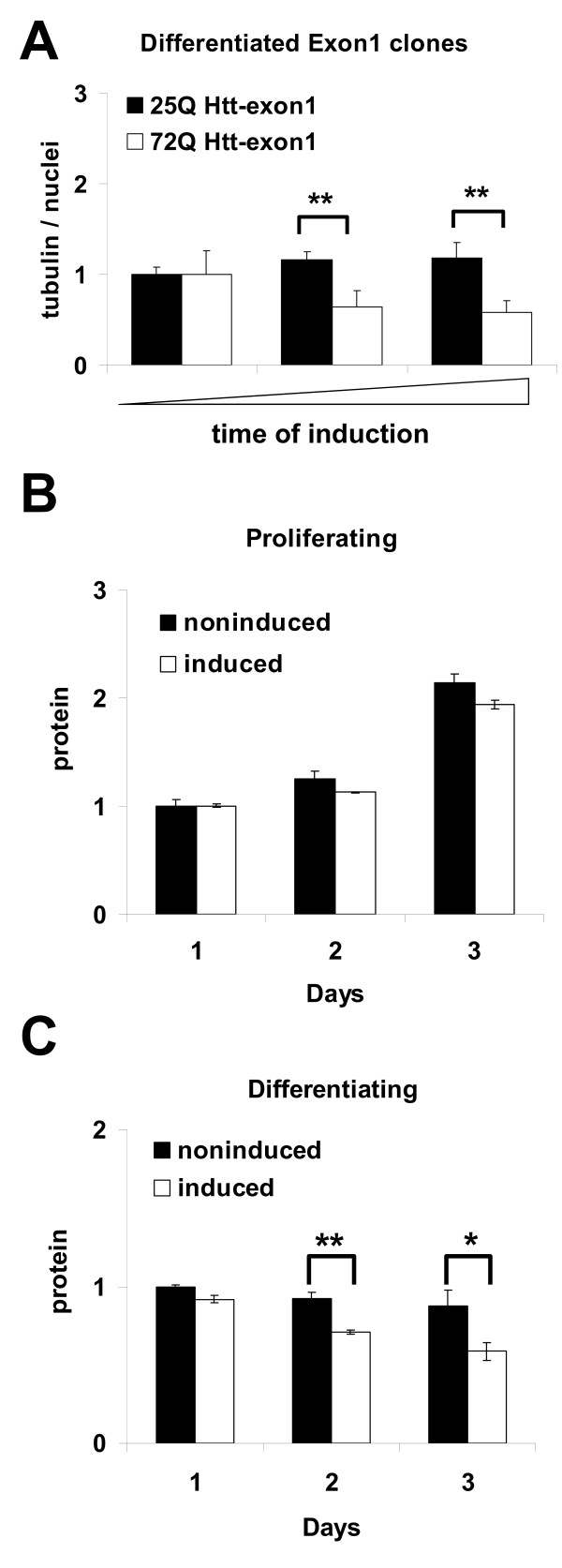
**Toxic effect of mutant huntingtin expression in differentiated cells**. **A**: Quantification of neurite extension as a function of cell number using InCell Western determination of tubulin versus nuclear ratios in differentiated HN10 lines one and two days after induction of wild-type (black bars) and mutant (white bars) Htt-exon1 with 500 nM RSL1. **B**, **C**: Total cell protein determinations in the presence or absence of 72Q Htt-exon1 expression in proliferating (**B**) or differentiated (**C**) HN10 cells. All graphs: n = 3, * p < 0.05, ** p < 0.01.

Upon differentiation, HN10 cells form a dense network of processes (see above). It is possible to determine automatically the extent of neurite outgrowth by measuring the total amount of tubulin in each well. Normalization to cell number was achieved by staining the cell nuclei and thus the extent of the neurite network is reported as the ratio of neurites (tubulin) per cell (nuclei) for each culture conditions. When compared to non-induced HN10 cells, 72Q Htt-exon1 expressing cells showed a significant decrease in the neurite network. Again, induction of 25Q Htt-exon1 showed a protective effect, since wild-type Htt expression resulted in more tubulin positive neurites per cell nuclei (Fig. 6A). In contrast, induced expression of 72Q Htt-857 had no significant influence on the neurite network (data not shown).

As a generic readout for cell viability, changes in total cellular protein upon 72Q Htt-exon1 expression were determined. Cellular protein content increased with time when culturing the cells under proliferating conditions, reflecting the mitotic nature of the cells. A small but not significant decrease in cellular protein levels was detected in the proliferating clones after 3 days expression of 72Q Htt-exon1 compared to proliferating non-induced clones (Fig. 6B). Toxicity of 72Q Htt-exon1 became more prominent when keeping the clone under differentiating, post-mitotic conditions (Fig. 6C). Here, significant decrease of cellular protein levels between non-induced and induced cells were detected already after two days of 72Q Htt-exon1 expression and this effect became more pronounced after 3 days of induction, indicating a general impairment of cell viability as a result of mutant Htt expression in differentiated cells.

## Discussion

In order to establish a cellular model that can be used for drug discovery and ultimately to elucidate some of the pathogenic mechanisms leading to HD, we developed a flexible neuronal cell line system where expression level and exposure time of mutant Htt can be accurately controlled.

In HN10 cells, mutant Htt toxicity was most evident when the cells became post-mitotic. The ability to culture the cells under proliferating as well as differentiating conditions is relevant considering that terminally differentiated cells such as neurons and myotubes display the strongest aggregate formation and are most vulnerable to mutant Htt expression [34,35,46-48]. Since the amount of mutant Htt directly impinge on its toxicity [49], when cell division is stopped, intracellular Htt may surpass the threshold concentration required to initiate a cascade of toxic events ultimately compromising neuronal activity and survival. This may explain the results obtained when comparing dividing and non-dividing HN10 cells. Firstly, we demonstrated accumulation of mutant Htt leading to significant load of intranuclear inclusions. Then, we identified early abnormalities changes in transcription in differentiated and dividing HN10 cells. Finally, we show clear Htt toxicity phenotypes in post-mitotic cells such as aconitase impairment, caspase 3 activation, neurite loss and reduced total cell protein indicating an ongoing neurodegenerative process. Further studies are required to identify and address in more details the specific cellular pathways involved in Htt toxicity.

By comparing the effects of 25Q Htt-exon1 and 72Q Htt-exon1 expression on cell viability, we showed that the sensitivity of differentiated HN10 cells to mutated Htt is specific to the elongated polyglutamine stretch in the Htt fragment and not a cause of ectopic expression of a short Htt fragment. Interestingly, the expression of wild-type Htt resulted in a protective effect on caspase 3 activity, a finding which is in agreement with earlier *in vitro *and *in vivo *evidence assigning a neuroprotective role to normal Htt [45,50,51]. The use of an unmodified Htt fragment instead of Htt fusion proteins broadly used in previous investigations may highlight more disease-relevant phenotypes. Similarly, conditional Htt expression may reduce the risk of possible clonal artifacts, which are often encountered when comparing different clones generated from the same parental cell line.

While the expression of amino-terminal mutated fragments is known to lead to increased Htt toxicity, our model prevents dissecting the cellular pathways regulating proteolytic processing of full-length Htt. In addition, it is not excluded that additional pathogenic mechanisms are initiated by more carboxy-terminal regions of the mutated Htt protein. As an example, the nuclear export signal present at the carboxy-terminus of Htt has been implicated in Htt toxicity [52]. Hence, further developments of our models will include the generation of inducible full-length Htt cell lines.

## Conclusion

In summary, our HN10 cell-based neuronal model displayed several disease-relevant features typical of HD, such as Htt aggregation, the characteristic transcriptional abnormalities, decreased cell viability, impaired mitochondrial function and neurodegeneration [53]. Because of the possibility to dissect these features in a time-dependent as well as huntingtin expression level-dependent manner, these cell lines represent a viable HD *in vitro *model for drug discovery.

## Competing interests

Andreas Weiss, Ana Roscic and Paolo Paganetti are employed by Novartis Pharma AG, Basel, Switzerland.

## Authors' contributions

AW carried out the experiments, planned the design of the experiments and drafted the manuscript. AR performed and analyzed the subcellular fractionation experiment. PP conceived the study, participated in its design and coordination and drafted the manuscript. All authors read and approved the final manuscript.
